# Bone-Targeted Nanoparticle Drug Delivery System: An Emerging Strategy for Bone-Related Disease

**DOI:** 10.3389/fphar.2022.909408

**Published:** 2022-05-31

**Authors:** Yulin Chen, Xianmin Wu, Jiadong Li, Yingying Jiang, Ke Xu, Jiacan Su

**Affiliations:** ^1^ Institute of Translational Medicine, Shanghai University, Shanghai, China; ^2^ School of Medicine, Shanghai University, Shanghai, China; ^3^ School of Life Sciences, Shanghai University, Shanghai, China; ^4^ Department of Orthopedics, Shanghai Zhongye Hospital, Shanghai, China

**Keywords:** bone targeted, drug delivery, bone diseases, nanoparticles, stimulus-responsive

## Abstract

Targeted delivery by either systemic or local targeting of therapeutics to the bone is an attractive treatment for various bone metabolism diseases such as osteoporosis, osteoarthritis, osteosarcoma, osteomyelitis, etc. To overcome the limitations of direct drug delivery, the combination of bone-targeted agents with nanotechnology has the opportunity to provide a more effective therapeutic approach, where engineered nanoparticles cause the drug to accumulate in the bone, thereby improving efficacy and minimizing side effects. Here, we summarize the current advances in systemic or local bone-targeting approaches and nanosystem applications in bone diseases, which may provide new insights into nanocarrier-delivered drugs for the targeted treatment of bone diseases. We envision that novel drug delivery carriers developed based on nanotechnology will be a potential vehicle for the treatment of currently incurable bone diseases and are expected to be translated into clinical applications.

## 1 Introduction

Bone is one of the essential organs of the human body, which is composed of 60% inorganic minerals and 30% organic matrix, as well as 10% cells and blood vessels ; the inorganic minerals are generally known as hydroxyapatite (HA) (Ca_10_(PO_4_)_6_(OH)_2_), while the organic matrix includes collagens, proteoglycans, and lipids (Shea and Miller, 2005; Chen et al., 2022). The skeleton performs many functions in the body; one is that it supports the body structure, while the other is that it gives protection to the organs inside the structure and also acts as the storage of minerals and involves in the production of blood (Florencio-Silva et al., 2015). The highly specified dynamic tissue is constantly being metabolized and remodeled throughout life to maintain a healthy skeletal structure for these functions. Bone metabolism involves multiple basic bone cells that act as key regulators, including osteocytes, osteoblasts, and osteoclasts, which either on their own or in interaction keep the balance between bone catabolism and anabolism (O'Brien et al., 2013; Gao et al., 2021). It begins with osteocytes stimulated by mechanosensory stimulation to initiate bone remodeling, recruiting osteoclasts to the old or damaged bone surface which promote bone resorption (the dominant event in the second phase), the mesenchymal stem cells and bone progenitor cells are recruited to the site at the same time, followed by MSCs differentiating into osteoblasts in the third phase to mediate the bone formation for a sustained time, and finally end with the mineralization of the organic matrix called osteoid to form new bones (Crockett et al., 2011). When cells or cytokines in any of these four phases are altered, it may result in bone metabolism diseases (Rodan and Martin, 2000; Li et al., 2022).

Common bone diseases include osteoporosis (OP), Paget’s disease, osteoarthritis (OA), osteosarcoma (OS), and osteomyelitis (Xue X. et al., 2021). These diseases are a public health problem that cannot be ignored as it negatively affects the regular functions of the skeleton, not only causing great suffering to the patient which limits them from living a normal life but also putting an enormous burden on the health care system (Harvey et al., 2010; Kansara et al., 2014; Fang et al., 2021). With great progress in bone biology research, there are currently several different kinds of drugs available for therapeutic interventions, but it is difficult to release the medicine into the tissue after an oral or intravenous injection and most of it will be excreted from the body before reaching the bone because of the dense and occluded characteristics of the pathologic skeletal tissues, which usually require higher or more frequent drug doses to ensure the therapeutic effect (Hirabayashi and Fujisaki, 2003; Stepensky et al., 2003). However, it is likely that higher drug concentrations may also have toxic effects on other organs and cause a series of adverse reactions (Khosla and Hofbauer, 2017). Therefore, the focus of the present research is to develop well-targeted, hyperpermeable, sustained release, and less-toxic bone-targeted drug delivery systems (Cheng et al., 2017).

In the past decades, for bone-targeting drug delivery, researchers have proposed to use various nanomaterials as carriers, such as polymeric nanoparticles, liposomes, micelles, vesicles, dendritic macromolecules, and scaffolds (Shuai et al., 2020; Cai et al., 2021; Zou et al., 2021; Liu et al., 2022b). Nanomaterials usually have unique structures with adjustable size, shape, and surface properties that have a crucial impact on drug loading and release, cellular uptake, and blood circulation metabolism, which have displayed the benefits of high loading capacity, excellent biocompatibility, and ease of various surface modifications for use in drug carriers (Singh et al., 2019). For example, the broadly studied inorganic material mesoporous silica nanoparticles (MSNs) have shown great applicational advantages in antitumor therapy with good stability, high drug loading, and high degree of customization for applicability (Baek et al., 2015). The most common strategy is to use the unique hydroxyapatite component of the bone, the pharmacokinetic profile can be significantly enhanced and potentiate the skeletal deposition of the drug by combining bone affinities with therapeutic agents (Raina et al., 2020). Since 1986, when the concept of “bone targeting” was put forward, bone targeting research has advanced considerably (Pierce and Waite, 1987). In 1999, there was a study that demonstrated the unique properties of bisphosphonate drugs for targeting organ and binding to the bone matrix (Porras et al., 1999). In the meantime, the application of nanotechnology for drug delivery can not only improve the drug loading capacity, but also the stability, enable a sustained and controlled release of drugs, prolong the retention time of drugs in the body, and reduce the toxicity of drugs (Choi and Kim, 2007). As mentioned previously, these nanotechnology-based bone-targeting drug delivery strategies have shown great potential for bone metabolic diseases.

In this review, we will summarize the bone-targeting approaches and specific applications for therapeutic bone diseases. First, we introduce the bone-targeting strategies, which are essential for constructing bone-targeting nanomaterials, we then conclude the applications of bone-targeting nanomedicines in bone metabolic diseases, and make a discussion of these delivery strategies, and finally, we propose a prospect for the research directions and application prospects of nanomedicines for bone-related diseases in the future.

## 2 Bone-Targeted Strategies

**FIGURE 1:**
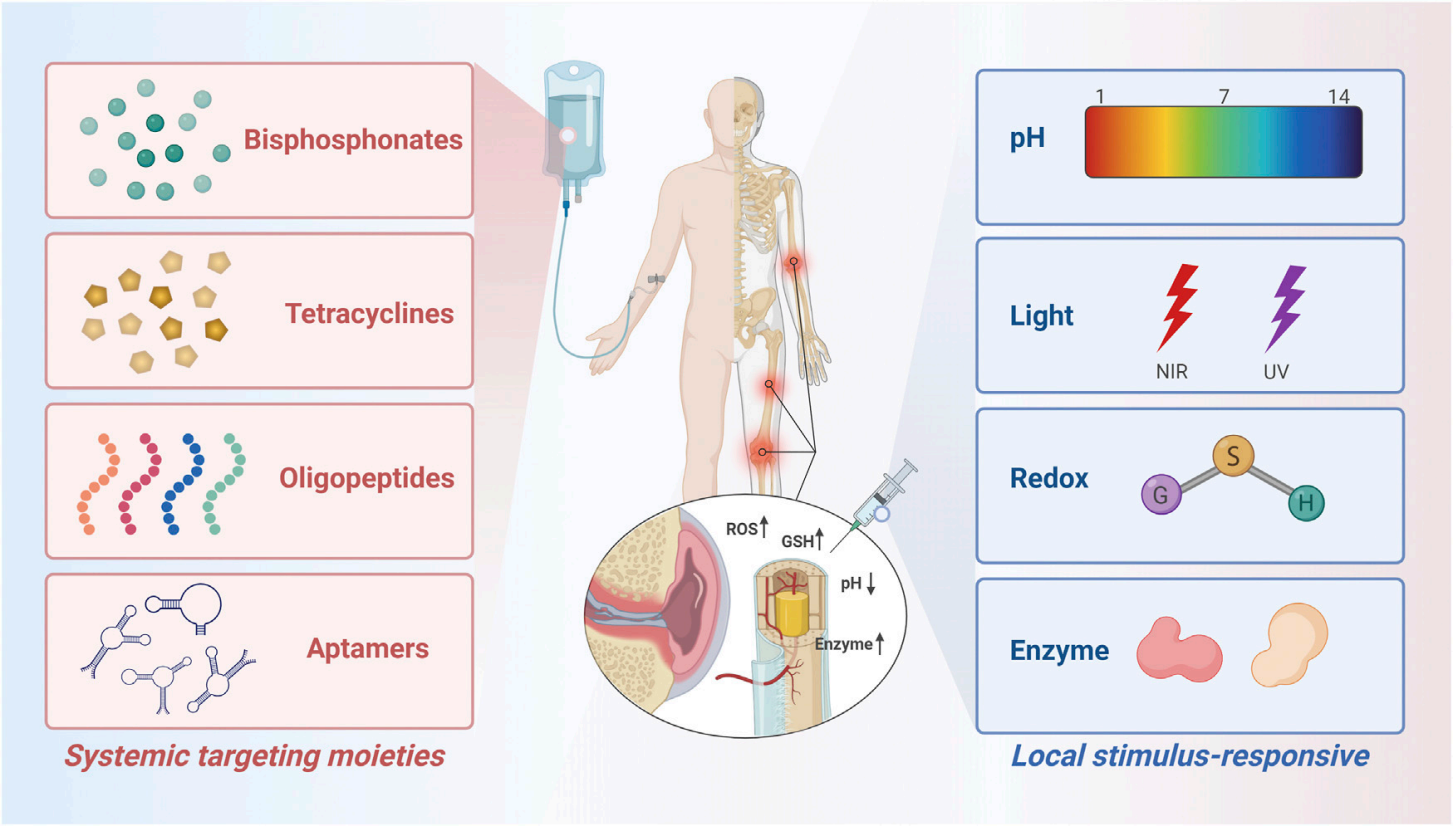
Schematic diagram of the molecules and groups used to modify the bone-targeted nanoparticle drug delivery system. Created with BioRender.com.

There are two main types of bone-targeting strategies commonly used today, which we classify as systemic targeting and local targeting (Figure 1). Systemic targeting is often achieved through tail vein injection of a drug delivery system, where the target molecule binds to the hydroxyapatite in the bone and delivers the drug to the bone for deposition to the lesion (Wang et al., 2005). Bone-seeking moieties used in this method may include bisphosphonates, tetracyclines, acidic oligopeptides, and aptamers (Table 1), which have been validated and developed in numerous studies and are sufficient to treat most diseases including bone-related diseases (Perrin, 1965; Fujisawa et al., 1996; Nimjee et al., 2005; Ossipov, 2015). However, bone diseases are complex and diverse, and some of them may not be suitable for targeted HA therapeutic strategies (Kargozar et al., 2020). With the proposed concept of “microenvironment”, the focus on targeted and sustained therapeutic agents has gradually shifted to stimulate drug release in recent decades (Mura et al., 2013; Baek et al., 2015). To accommodate weak acidity, abnormal enzyme and redox levels, and localized heat and swelling in the pathologically altered bone microenvironment, more precise and sensitive response components are needed to achieve on-demand, targeted drug release, thereby minimizing the possibility of an abrupt or premature drug release and reducing the likelihood of adverse effects (Lavrador et al., 2018; Hopkins and Qin, 2020; Zhang et al., 2022). Scientists have developed some novel local targeting systems over the past years, i.e. stimulus-responsive drug delivery systems (Table 2). The drug will be released at the target site only when stimulated so that it enters the bone at an effective concentration (Xiong et al., 2019b). To a large extent, the development of smart nanocarriers responsive to bone microenvironment stimuli will accelerate the progress of bone disease treatment in the future.

**TABLE 1:** Summary of systemic bone-targeting moieties.

Agents	Targeting site	Limitations	References
Bisphosphonates	HA	Adverse effects such as osteonecrosis of the jaw, atrial fibrillation, and gastrointestinal ulceration; Long-term deposition in the skeletal tissue	Watts and Diab, (2010)
			McClung et al. (2013)
Tetracyclines	HA	Low affinity for pathological skeletal sites with severe bone loss; Tooth staining, decreased tooth hardness, and damage to tooth enamel	Tam and Anderson, (1980)
			Peterson, (1984)
Oligopeptides	HA	Low affinity to the target site relatively leads to the possibility of off-target; tendency to aggregate; and requires special storage conditions to maintain stability	Sekido et al. (2001)
			Cheng et al. (2017)
Aptamers、signaling molecules	Cells (or cell surface receptors)/signaling pathways	Poor *in vivo* stability; immunogenic; heterogeneity may exist at different sites	Ye et al. (2012)
			Wang et al. (2021)

**TABLE 2:** Summary of stimulus-responsive strategies.

Stimulus	Stimulire-sponsive linkages/groups	Limitations	References
pH	Hydrazone, amide, acetal ketone, carboxylic acid, sulfonic acid, amino, pyridine, imidazole	Lack of toxicity data; Low mechanical strength; the lesion location may be similar to the surrounding normal tissue	Pang et al. (2016)
Light	Azobenzene, spiro-pyran, diaryl ethylene, triphenylmethane, azidonaphthoquinone, cinnamic acid ester, coumarin	May cause tissue temperature to rise and damage the tissue; safety and/or biodegradability require further verification	Dvir et al. (2010)
Redox	Disulfide bond (S-S), diselenide bond (Se-Se)	Sensitivity is influenced by the ROS or GSH concentrations at different sites	Liu et al. (2020)
			Ren et al. (2021)
Enzyme	Phospholipase, oxidoreductase, protease, glycosidase, lipase	Possible hydrolysis; Not clear whether it causes side effects	Chen et al. (2021)

### 2.1 Systemic Bone-Targeted Strategies

#### 2.1.1 Bisphosphonate

Bisphosphonates (BPs) are a class of compounds that are broad and efficiently used in the treatment of bone metabolism-related diseases, as they inhibit osteoclast differentiation and decrease bone resorption (Coxon et al., 2006). The function of BPs largely depends on the backbone structure of the two terminal phosphate groups bound to the central carbon atom (P-C-P), which can chelate with Ca^2+^ through electrostatic interactions and confers a binding affinity to HA (Russell, 2007). Two covalent side chains, R1 and R2, can be modified such that it may affect the affinity and pharmacological activity of BPs, for example, N–BPs obtained by nitrogen modification of the R2 side chain (e.g., alendronate, zoledronate, ibandronate, etc.) show higher bone affinity compared with non-nitrogenous BPs, while non-nitrogenous BPs will metabolize to ATP analogs that are cytotoxic and cause osteoclast apoptosis, while N–BPs inhibit the bone resorption function by reducing osteoclast activity, but they both result in slowing bone loss in the end (Lin, 1996; Russell, 2007; Ebetino et al., 2011). Moreover, the P-C-P chemical structure makes bisphosphonates resistant to chemical and enzymatic hydrolyses and can be deposited in the bone for the long term (which also depends on the patient’s bone conversion rate and renal function) (Russell et al., 2008). Studies have shown that in the treatment of BPs, some of the drugs undergo a process of binding to the bone and then releasing and re-binding to the bone. However, the ability of it to resist bone resorption does not improve with time (Watts and Diab, 2010). Although bisphosphonates have been used in clinical treatments for many years, BPs still have adverse effects such as osteonecrosis of the jaw, atrial fibrillation, gastrointestinal ulceration, etc (McClung et al., 2013).

Moreover, with the direct use of BPs as therapeutic agents, one can selectively deliver drugs to the bone by combining them with nanoparticle drug delivery systems. Previous studies have demonstrated that nanoparticles composed of alendronate (Aln) as a targeting agent, co-modified with the hydrophilic component of poly (oxyethylene) (PEG) to the surface of poly (lactic-co-glycolic acid) (PLGA), an FDA-approved and commonly used drug delivery system, had excellent and specific adsorption to HA, and the NPs can also be loaded with estrogen as a therapeutic for osteoporosis, which avoids estrogen acting on tissues other than the bone that may cause side effects like intrauterine hemorrhage or even endometrial and breast cancers (Choi and Kim, 2007). Subsequent studies have also performed hemocompatibility and cytotoxicity studies on these PLGA–ALE NPs, and the results confirm that NPs may be considered suitable for intravenous administration (Cenni et al., 2008), given that nanodiamonds (NDs) have an excellent alkaline phosphatase (ALP) activity and can enhance the proliferation and differentiation of osteoblasts. Ryu et al. modified NDs with oleic acid to obtain nanoparticles with good dispersion properties, and then conjugated them with alendronate through carboxyl groups on the surface of NDs to form Aln-NDs. Compared with the unmodified NDs, the Aln-NDs showed preferential affinity for osteoblasts, and the ALP activity was 2.2 folds higher than that of the Aln group and 1.6 folds higher than that of the NDs group after 7 days. The results of the *in vivo* experiments showed that nanoparticles are highly aggregated in bone tissue and can be used as a bone-targeted drug carrier for osteoporosis treatment, which opens the door for the research of nanomaterials for osteoporosis treatment (Ryu et al., 2016). Enhancing drug half-life and reducing the possibility of off-targeting are essential objectives in the development of bone-targeted nanosystems, Hoque et al. modified hyaluronic acid methacrylate (HA-MA) by introducing Aln molecules via coupling reaction, and the obtained nanocarriers were loaded with adenosine molecules by dialysis. *In vivo* imaging system (IVIS) results showed that there was higher aggregation in bone tissue after systemic administration of Aln-ND, and it had a therapeutic effect in promoting bone formation and delaying bone loss in an ovariectomized model of osteoporotic mice (Hoque et al., 2021).

#### 2.1.2 Tetracycline

Tetracycline, discovered in the 1940s, is a broad-spectrum antibiotic drug (Kunin, 1968). Tetracycline has the effect of inhibiting bacterial growth at high concentrations and has been used extensively in prevention and treatment of infections in humans and animals (Nguyen et al., 2014). In 1957, one study noticed that tetracycline showed the ability to bind rapidly and specifically to the bone for a considerable period after administration, it could be deposited in the bone tissue and incorporated into the new bone (Milch et al., 1957). Later, tetracycline was developed as a target labeling vehicle due to its fluorescence under UV light (Tam and Anderson, 1980). The osteoaffinity of tetracycline depends on whether it can be complexed with hydroxyapatite in the bone. Spectroscopic experimental data indicated that the phenolic *ß*-diketone group is attached to carbons 10, 11, and 12 playing a major role in forming complexes with calcium and other metal ions (Perrin, 1965; Shea and Miller, 2005). Due to the high affinity of tetracycline for HA, it should be avoided in pregnant or lactating women, and developing children, because it might cause permanent tooth staining and may lead to decreased tooth hardness and damage to tooth enamel (Sánchez et al., 2004).

**FIGURE 2:**
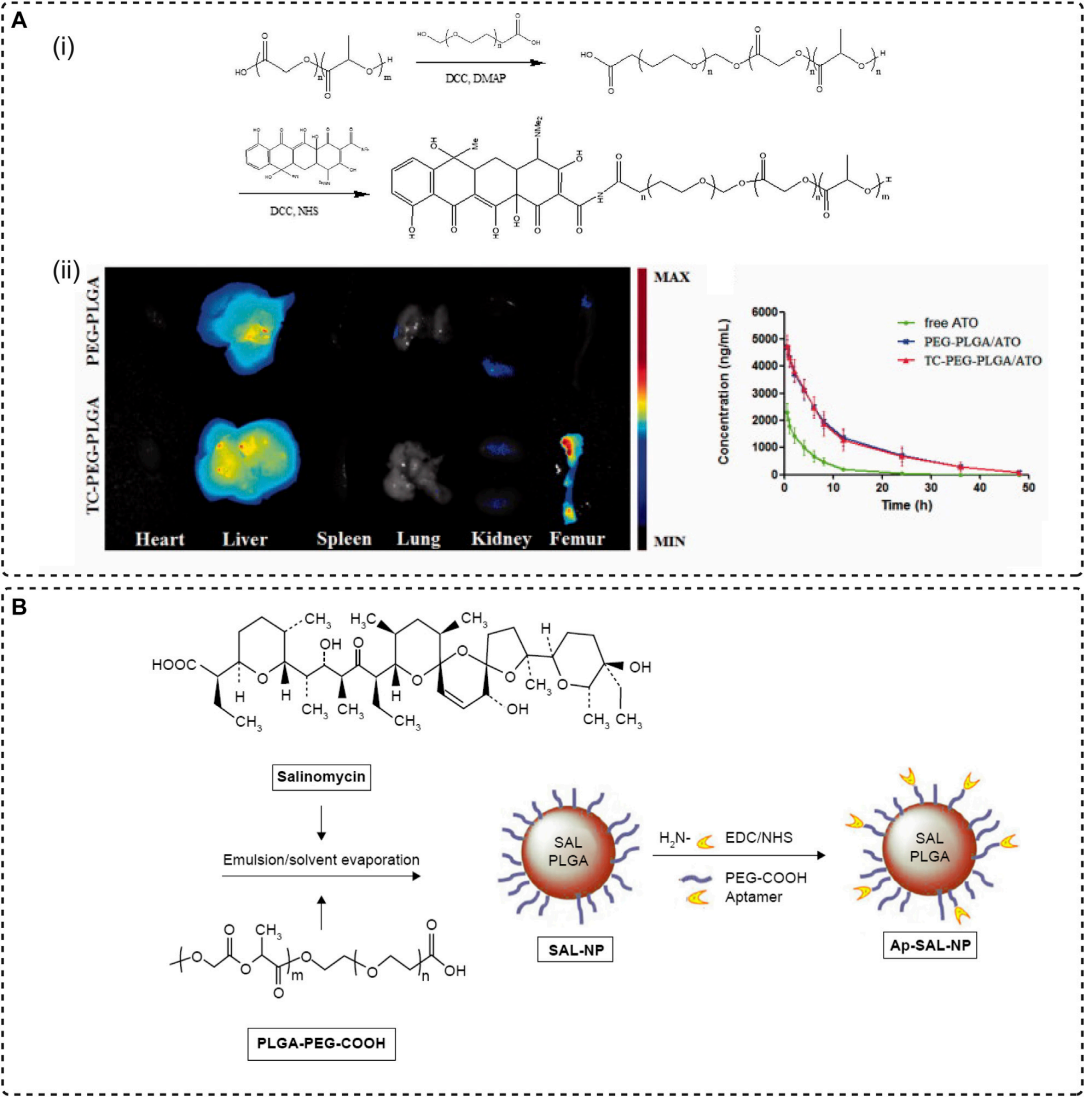
Systemic targeting strategies. **(A)** Tetracyclines bind to the bone. **(i)** Synthesis of TC-PEG-PLGA. **(ii)** Modification of TC mediates the enrichment of drug-loaded micelles in the femur (Xie et al., 2017), Copyright 2017, TAYLOR & FRANCIS LTD. **(B)** The preparation procedure of the drug delivery system (Ap-SAL-NP) by using CD133 as an aptamer and conjugating with salinomycin-loaded PEGylated PLGA nanoparticles (Ni et al., 2015), Copyright 2015, Dove Medical Press.

Neale et al. synthesized a novel bone-targeting medicine which was modeled after the tricarbonyl methane grouping of ring A of tetracycline, then conjugated with estradiol resulting in a bone-targeted estrogen (BTE2-A1) that showed an increase in its ability to bind to HA. The pharmacological and toxic effects of the osteotropic estradiol delivery system were evaluated in the OVX rat model and showed positive results (Neale et al., 2009). Another attempt to synthesis the amphiphilic copolymer PEG–PLGA micelles modified with TC to encapsulate hydrophobic atorvastatin (ATO) for the targeted treatment of osteoporosis. Mice femur showed strong fluorescence after a 24 h intravenous injection of fluorescent probe Dir-loaded micelles, and after 12 weeks of treatment, the femur BMD (bone mineral density), a critical parameter for evaluating fracture risk in OVX rat receiving TC-PEG-PLGA/ATO micelles, exhibited significant therapeutic efficacy (Figure 2A) (Xie et al., 2017). Fan’s team combined the traditional bone-affinity agent tetracycline with a novel concept of smart response to develop a tetracycline surface-functionalized nanoliposome for encapsulating the alkaline compound sodium bicarbonate (NaHCO_3_). The introduction of tetracycline-enriched NaHCO_3_ on the bone surface in large amounts counteracted the acidification of the bone microenvironment caused by osteoclasts with an acid-base neutralization strategy, thereby postponing osteoporosis (Lin et al., 2020).

#### 2.1.3 Oligopeptides

Given that hydroxyapatite, which is not present in soft tissues, exists as a specific component in bones and teeth, targeting HA may be a promising method for selective bone-targeted drug delivery (Oliveira et al., 2017; Rotman et al., 2018). Several results demonstrate that non-collagenous proteins in the bone matrix (bone sialoprotein and osteopontin, etc.) have affinity to hydroxyapatite and affect osteogenic mineralization. These proteins have repetitive acidic amino acid sequences of l-aspartic acid (L-Asp) and l-glutamic acid (L-Glu) (Oldberg et al., 1986; Butler, 1989; Rotman et al., 2018). Previous research has established that the affinity of the peptides for HA will be increased when there are repeated Asp or Glu units in the amino acid sequence. For instance, Kasugai et al. designed a conjugated (Asp)6 to fluorescein isothiocyanate (FITC). FITC was unable to bind to HA, however, fluorescence was not observed in any tissues other than bones and teeth at 24 h after systemic administration of (Asp)6-FITC into rats (Kasugai et al., 2000). In another work, a novel pro-drug constructed by estradiol conjugated with L-Asp-hexapeptide showed a potent anti-osteoporotic therapeutic effect in OVX mice (Yokogawa et al., 2001).

Liposomes are the only drug delivery system currently approved by the FDA for clinical application. In 2012, researchers designed an osteogenic siRNA delivery system to specifically target bone-formation surfaces, which is (AspSerSer)6 linked with a DOTAP-based cationic liposome. The resultant liposomes were bound to the bone-forming surface more than to the bone-resorbing surface. Targeted delivery resulted in greater enrichment of siRNA in osteoblasts, which promoted bone formation in OVX rats by intervening in bone anabolism (Zhang et al., 2012). Alternatively, a five-amino acid motif oligopeptide Ser-Asp-Ser-Ser-Asp (SDSSD) was obtained via phage display screening technique, which was directly and specifically targeted to osteoblasts by binding to periostin, and then is conjugated to polyurethane (PU) surfaces to obtain a nanomicellar vector. The SDSSD-PU complex was loaded with siRNA/microRNA by electrostatic interaction and showed a superior bone targeting delivery ability in both *in vivo* and *in vitro* experiments (Sun et al., 2016).

Together, these studies indicate that modifying nanoparticles for targeted delivery using acidic oligopeptides as bone-seeking agents has great prospects. Moreover, compared with peptides and other proteins, oligopeptides have the advantages of high stability, good tissue permeability, and low immunogenicity due to their lower molecular weight, and compared with BPs, which have a short half-life and can be metabolized to non-toxic substances *in vitro* (Sekido et al., 2001; Cheng et al., 2017).

#### 2.1.4 Others

There are several other molecules that can be used to target bones apart from those mentioned previously.

Conceptually, aptamers are a class of single-stranded DNA/RNA oligonucleotide molecules with high affinity and strong targeting characteristics similar to antibodies (Nimjee et al., 2005). However, unlike antibodies, aptamers are produced through a selection process known as the Systematic Evolution of Exponentially Enriched Ligands (SELEX) *in vitro* which is chemically synthesized and hence can be specifically modified in structure to bind to specific targets in a complementary form as one prefers (Sefah et al., 2010). It has been widely used to recognize various targeted sites, such as small molecules of antibiotics, short peptides, metal ions, and organic dyes, as well as a wide variety of proteins with complex multimeric structures, also including cells, viruses, and bacteria (Wang et al., 2015). Aptamers have been used for diagnosis, detection, and targeted therapy due to their easy acquisition and great targeting ability (Chinnappan et al., 2021). In addition, the high stability of aptamers, their low toxicity and immunogenicity, and their chemical modification to confer controlled or periodic denaturation and renaturation have expanded the flexibility of aptamer use in various biomaterials (Ye et al., 2012). One study by Ni et al. designed a drug delivery system (Ap-SAL-NP) for the targeted treatment of osteosarcoma by using CD133, a cancer stem cell (CSCs) marker for osteosarcoma, as an aptamer and conjugating with salinomycin-loaded PEGylated PLGA nanoparticles (Figure 2B). *In vitro* and *in vivo* demonstrate that aptamer-modified NPs not only have a specific killing effect on CD113^+^ osteosarcoma cells, but also have a targeted therapeutic effect on osteosarcoma xenograft mice (Ni et al., 2015).

Recently, a large and growing body of studies has demonstrated that biomimetic delivery vehicles have shown great potential for drug delivery, targeted therapies, and bioimaging (Meyer et al., 2015; Fang et al., 2018). As naturally derived nanoparticles, the membrane surface of exosomes contains transmembrane and membrane-anchored proteins that may enhance endocytosis, which facilitates the delivery of contents in terms of drug delivery (Wu et al., 2020; Liu et al., 2022a). The chemical composition and membrane structure of exosomes exhibit a similar biological property to biological surfaces and, in some cases, even have an innate targeting ability (Jiang et al., 2022). For example, Song et al. published an article on bone-targeting delivery via vascular endothelial cell (EC)-derived exosomes (EC-Exos). EC-Exos loaded with a fluorescent probe Dil was injected into mice by tail vein injection, and a clear fluorescent signal was observed in the skeleton after 8 h. Detection of differentially expressed proteins in different exosomes by mass spectrometry had a higher expression of pregnancy zone protein (PZP) in EC-Exos relative to other exosomes of bone-associated cell origin, which means PZP probably has a proactive effect on the bone targeting feature of EC-Exos. In addition, EC-Exos is biocompatible *in vivo*, demonstrating further use as a nanocarrier for the delivery of different therapeutic agents to the bone tissue (Song et al., 2019). Alternatively, we can improve their targeting by genetically engineering modifications. Several studies have revealed that the relationship between C-X-C motif chemokine receptor 4 (CXCR4) to stromal cell-derived factor 1 (SDF1), and the high levels of SDF1 expression in bone marrow have a recruiting effect on CXCR4^+^ hematopoietic stem cells (HSCs) and promote bone metastasis of CXCR4^+^ tumor cells. Based on the aforementioned, Hu et al. conducted a series of trials in which they genetically engineered NIH-3T3 cells to highly express CXCR4, and extracted CXCR4-expressing exosomes that were hydride with liposomes bearing antagomir-188 to obtain bone-targeting nanoparticles with the ability to modulate bone metabolism. The hybrid NPs showed a significantly higher bone mass reservation in OVX mice which is a prospective concept for the treatment of age-related bone loss (Hu et al., 2021b).

### 2.2 Local Responsive Bone-Targeted Strategies

#### 2.2.1 pH Response

Among the various responsive materials, pH-sensitive materials have generated research interest mainly owing to their relevance to the specific endogenous stimuli (Xiong et al., 2019a). The delicate changes in environmental and physiological pH values have an important influence on human health. Healthy tissues have a pH of approximately 7.4, while under pathological conditions, most tissues show a decrease in pH, such as inflammatory tissues (pH = 6.5) and tumor sites (pH = 6.5–7.2), and especially, the lysosomes of bone tissue cells can even reach below 6 (Pang et al., 2016). These differences in the microenvironment provide a pre-requisite for regional delivery or targeted treatment with pH-responsive nanocarriers. When the pH reaches a certain critical value, it will trigger intermolecular forces such as electrostatic interaction, hydrogen bonding, or covalent bonding on the nanocarrier to release the drug (Lavanya et al., 2020).

**FIGURE 3:**
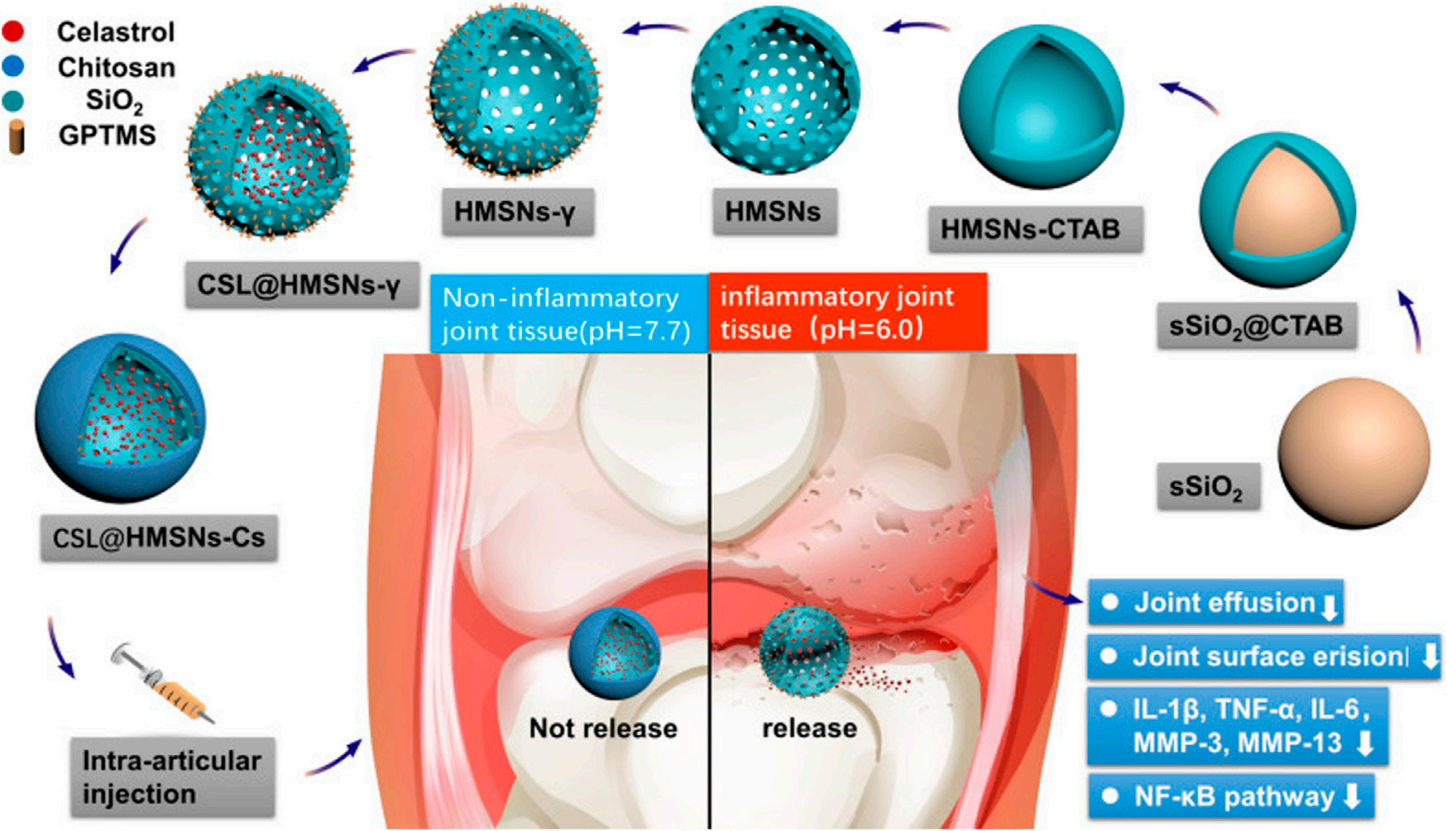
Synthetic route of CSL@HMSNs-Cs and the molecular mechanism of the local injection for OA treatment. Firstly, sSiO_2_ nanocore was constructed by the excellent Stöber method, and ethyl orthosilicate was covered on the surface by surfactant CTAB, followed by etching out the hollow structure of nanoparticles with aqueous sodium salt solution, removing CTAB and obtained hollow MSN, and chitosan encapsulation after celastrol diffusion into nanoparticles. The acidic environment in the arthritic joint cavity stimulated chitosan collapse, thus releasing the drug, inhibiting the NF-κB signaling pathway, reducing the expression level of inflammatory factors, and improving the pathological manifestation of osteoarthritis (Jin et al., 2020), Copyright 2020, Springer Science and Business Media LLC.

Celastrol (CSL) is a drug extracted from Radix Rehmanniae and has been extensively used for OA treatment due to its potent anti-inflammatory and antioxidant efficacy (Cui et al., 2020). However, the drug toxicity and low solubility of CSL have limited its clinical application. Thus, Jin et al. designed a highly soluble pH-responsive nanomaterial medicine that used hollow mesoporous silica nanoparticles (MSN) as nanocarriers to carry CSL and encapped with chitosan to confer a pH-responsive property for intra-articular injection therapy of osteoarthritis (Figure 3). The CSL@HMSNs-Cs present a high biocompatibility and extraordinary therapeutic efficacy (Jin et al., 2020). In addition to the acidic environment of osteoarthritis, an acidic microenvironment also exists in osteoporotic joint sites. Dou et al. proposed a cerium oxide bone-targeted pH-stimulated nanomaterial for the bone resorption void microenvironment in response to acidification of mature osteoblasts (mOCs) (pH = 3–4). By controlling the surface Ce3^+^:Ce4^+^ ratio of the cerium nanosystem (CNS), they were guided into the acidic extracellular microenvironment, where the antioxidative nanoparticles were stimulated to convert to oxidative state further increasing the accumulation of intracellular ROS and calcium oscillations, which decreased the viability of mOCs significantly, preserved the anabolic capacity of preosteoclasts (pOCs), and resisted excessive bone loss in the treatment of osteoporotic ovariectomized mice (Dou et al., 2021).

Polymeric hydrogels are attractive tissue repair materials (Kurian et al., 2022). Biological scaffold materials constructed from hydrogels can interact with surrounding tissues, modulate the activity of cells and growth factors, and induce osteogenesis and angiogenesis (Siddiqui et al., 2021). Recently, Zhao and his co-workers prepared a hybrid nanoparticle (CMCh-ACP) by mixing carboxymethyl chitosan (CMCh) and amorphous calcium phosphate (ACP) and doping glucono *d*-lactone (GDL) into it by alkaline hydration. In this system, the change of pH on the surface of the hybrid nanoparticles can trigger the self-assembly of the hydrogel to form the scaffold, and ACP is a biological precursor of calcium phosphate, which can induce osteogenesis by adsorbing cells to the surface of the scaffold, and further enhance the osteogenic effect by promoting BMP9 (Zhao et al., 2019).

#### 2.2.2 Photoresponse

Light is a powerful stimulus in nature as a source of energy for living systems, has been extensively studied as an external stimulus for intelligent responsive materials (Bansal and Zhang, 2014). As early as 2006, Mayer and Heckel came up with the design concept of “optical switch” (Mayer and Heckel, 2006), which made it possible to construct photoresponsive systems by modifying various photochromic components, including photoisomerization, e.g., azobenzene, spiropyran (SP), and 2-diazo-1,2-naphthoquinone (DNQ), photocross-linking/-decross-linking, e.g., coumarin and cinnamoyl, photocleavage, e.g., coumarinyl ester, o-nitrobenzyl (ONB), and near-infrared (NIR) light (Dvir et al., 2010; Hansen et al., 2015; Zhou et al., 2018). Nanocarriers can either release cargos directly upon light stimulation or generate intermediate reactions that can produce intermediate signals/molecules (heat, reactive oxygen species (ROS), gas molecules, etc.) to promote cargo release by cascade conversion (Rapp and DeForest, 2021). Light as a type of electromagnetic wave, when irradiated on the photoresponsive material, is able to transfer some of its energy to the object, and the transferred energy accordingly triggers certain property changes such as chemical bonds, chemical groups, conformation, and polarity of the object, thus releasing or activating the carriers (Barhoumi et al., 2015; Fernandez and Orozco, 2021). It is easy to see that the light-responsive material has highly controllable characteristics of time, space, wavelength, and density, and using it as a drug delivery system can deliver the drug to the right location at the right time and maintain the therapeutic state for a certain time.

As we all know, chemotherapy is one of the most common methods to treat bone tumors, but it can also bring serious side effects along with the treatment. While NIR light-mediated photothermal therapy (PTT) and photodynamic therapy (PDT) can penetrate deep into the tumor site with almost negligible phototoxicity, which is a promising strategy to achieve precise bone tumor treatment (Markman et al., 2013), Tong with co-workers combined two anticancer drugs based on a thioketal (TK) linkage to synthesize pro-drugs, which were loaded in mesoporous silica nanoparticles modified with biphosphate moiety and the photosensitizer chlorin e6 (Ce6), to develop a pro-drug-loaded functional MSN for combined photodynamic therapy (PDT) and enhanced the chemotherapy effect for osteosarcoma. After the nanoparticles were uptaken by tumor cells, Ce6 was lasered to generate intracellular ROS, meanwhile, TK linkage was disrupted and DOX/DOXY were released at the bone tumor site sustainably to accelerate the production of ROS, triggered ROS burst, therefore leading to the enhancement of tumor cell inhibition and apoptosis (Tong et al., 2020).

The functions of NIR light in penetrating tissues, warm therapy, and targeted release can also be applied in osteoarthritis treatment. Xue et al. used hollow mesoporous copper sulfide nanoparticles (CuS NPs) as the core of a drug delivery nanosystem, modified with a composite membrane of neutrophil and erythrocyte membranes, for the delivery of dexamethasone sodium phosphate (Dexp). The nanoparticles were loaded with fluorescent probes, and *in vivo* fluorescence imaging demonstrated that the coverage of neutrophil membranes conferred the ability to target inflamed joints, and the introduction of erythrocyte membranes conferred the long retention effect of the nanoparticles. The therapeutic strategy of drug release from D-CuS@NR NPs in response to 1,064 nm NIR light reduced the expression of inflammatory factors in OA mouse joints and alleviated the damage to the cartilage matrix. The aforementioned findings suggest that this drug delivery system may be a new platform for the treatment of OA (Xue et al., 2022).

#### 2.2.3 Multi-Responses

Although the aforementioned single stimulus-responsive types can be used as a separate carrier for controlling drug release, multi-stimulus-responsive drug vehicles are becoming a research hotspot appearing in recent years, in order to achieve better bone-targeting and flexibility to match the many influencing factors in the skeleton, such as pH/MMP, pH/redox, and pH/temperature (Han et al., 2015; Kalhapure and Renukuntla, 2018; Chen et al., 2019).

Matrix metalloproteinases (MMP) are critical regulators of changes in the bone and joint microenvironment (Chen et al., 2021). Lan et al. developed a pH/enzyme-responsive nano-micelle based on a poly (2-ethyl-2-oxazoline)-poly (ε-caprolactone) (PPL) core that was grafted with a specific collagen type II-targeting peptide and coupled with black hole quencher-3 (BHQ-3) via amide reaction to target articular cartilage and respond to metalloproteinases-13 (MMP-13). This nanoplatform was further employed as the carrier to load psoralidin (PSO) for protection against cartilage damage as a target nanotherapeutic agent for osteoarthritis (Lan et al., 2020).

Due to the changes in glutathione (GSH) levels caused by metabolic changes, we can design targeted drug systems using high gradient levels of GSH at tumor sites. Li et al. designed another reduction/pH dual responsive nanocarrier for osteosarcoma therapy. The NP-PTX-DOX synthesized by self-assembling micelles of PEGylated-PαLA copolymer mPEG-PαLA in water, which encapsulated paclitaxel (PTX) and doxorubicin (DOX) by electrostatic and hydrophobic interactions. The PαLA backbone containing disulfide bonds and carboxyl groups controls the targeted release of the drug in the reducing and acidic microenvironment, and the drug is enriched in the osteosarcoma tissue. The targeted nanosystem exhibits improved OS inhibition compared to the drug-treated control group and reduces the toxicity to normal cells due to the targeting effect (Li et al., 2020).

## 3 Biomedical Uses of the Bone-Targeted Drug Delivery System

### 3.1 Osteoporosis

Osteoporosis (OP) is a systemic metabolic bone disease characterized by reduced bone mass and abnormal bone tissue microstructure, leading to increased bone fragility and fracture susceptibility (Seeman and Delmas, 2006). Epidemiological data show that the number of people suffering from osteoporosis in China is on the rise with the aging of the population, which also results in increased medical expenses (Wang et al., 2009). According to its etiology, OP can be divided into two types: primary and secondary, of which primary is the more common type, including old age and post-menopause (Li et al., 2021). The case of postmenopausal osteoporosis, which is mainly due to estrogen deficiency, leads to bone resorption more than bone formation, resulting in bone loss and high conversion osteoporosis. Estrogen is frequently used to maintain bones’ mineral density. However, this therapy is associated with breast congestion and edema and, more importantly, with increased rates of endometrial hyperplasia and breast cancer (Prestwood et al., 1995; Black and Rosen, 2016). Therefore, to reduce the risk of these side effects and improve treatment results, the exploitation of bone-targeted therapies for osteoporosis is a popular research topic today.

**FIGURE 4:**
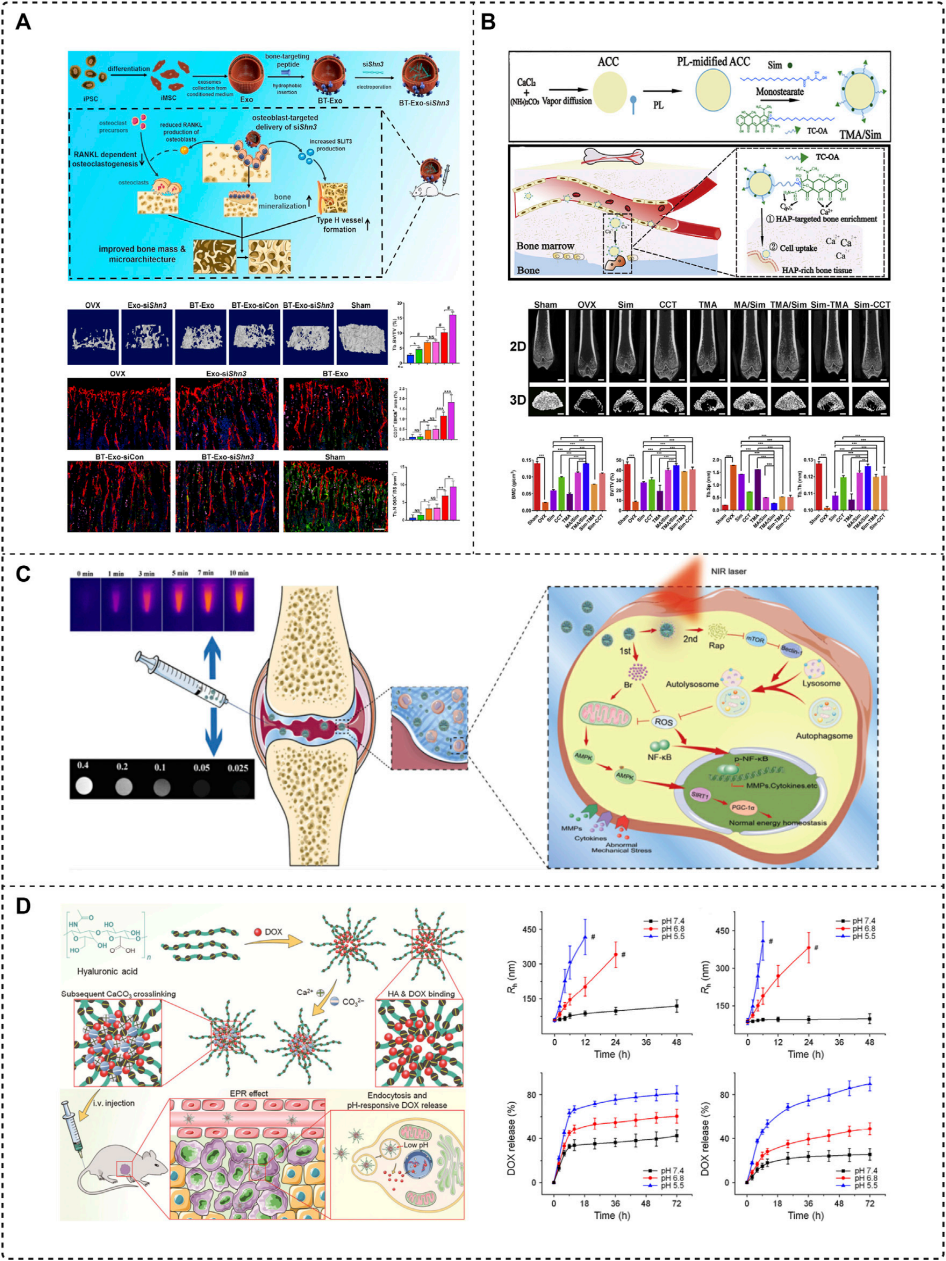
Application of the bone-targeted nanoparticle drug delivery system in bone diseases. **(A)** Bone-targeted engineered exosome platform BT-Exo-siShn3 enhanced osteogenic differentiation and promoted H-type vessel formation for OP treatment (Cui et al., 2022), Copyright 2022, Elsevier BV. **(B)** Scheme of the synthesis and mechanism of TMA/Sim, which provides calcium for bone structure (Tao et al., 2021), Copyright 2020, Elsevier BV. **(C)** Collagen-II targeting peptide and MOF-modified MPDA NIR-responsive dual delivery system (RB@MPMW) for rapamycin-targeted cartilage delivery in OA (Xue S. et al., 2021), Copyright 2021, Elsevier BV. **(D)** CaCO_3_ crosslinked HA nanoparticles to deliver DOX sensitivity to the acidic tumor microenvironment and release DOX for therapy of osteosarcoma (Zhang et al., 2018a), Copyright 2018, Springer Nature.

The application of gene therapy to bone metabolic diseases is still not translated into clinical applications owing to the absence of suitable targeted delivery systems to ensure the safety and efficacy of the treatment. In 2015, Liang et al. developed the first aptamer-functionalized liposome nanosystem, in which they selected osteoblast-specific aptamer CH6 by cell-SELEX, and the ligand-modified PEGylated liposome was mainly via macropinocytosis, to achieve the targeted release of the osteogenic Plekho1 siRNA, which promotes silencing of a negative regulator of bone formation genes in osteoblasts and increases bone formation, as evidenced by increased bone mass and improved bone microarchitecture in OVX rats (Liang et al., 2015). In addition, Cui et al. constructed an exosome-loaded Shn3 gene siRNA delivery system BT-Exo-siShn3 as a novel OP treatment (Figure 4A). The bone-targeting peptide was anchored to the exosome membrane by hydrophobic interaction modification, which conferred exosomes the ability to deliver siRNA to osteoblasts. The Shn3 gene-silencing reduced RANKL expression in osteoblasts and enhanced osteogenic differentiation, while inhibiting osteoclast activity, and prevented OVX-induced bone loss, promoted H-type vessel formation and bone mineralization (Cui et al., 2022). The aforementioned bone-targeting nanoparticles provide a forceful concept for the research of delivering siRNA to treat osteoporosis.

Calcium supplements are a clinically indicated agent for the basic therapy of osteoporosis, which can reduce bone loss and improve bone mineralization possibly, but typically requires high and repeated doses of administration, and the lack of targeting leads to poor treatment outcomes. Tao et al. reported an oral bone-targeted and OP microenvironment (water/pH) responsive carrier for *in situ* calcium supplements (Figure 4B). An amorphous calcium carbonate (ACC) platform was synthesized as the core skeleton of the drug delivery system (TMA), modified with tetracycline (Tc) and coated with monostearin (MS), further loaded with simvastatin (Sim) to construct a bone-targeted drug delivery system (TMA/Sim). Combining *in situ* calcium supplementation and targeted administration of simvastatin could deliver a promising OP therapy, which could be a hopeful therapeutic regime possibly (Tao et al., 2021).

### 3.2 Osteoarthritis

Osteoarthritis (OA) is a chronic arthropathy characterized by degenerative destruction of articular cartilage, local inflammation, subchondral bone sclerosis, and osteophytes, commonly diagnosed in the elderly (Hunter and Bierma-Zeinstra, 2019; Hu et al., 2021a). It is mainly due to the imbalance between the normal degeneration and formation of articular cartilage, extracellular matrix, and subchondral bone caused by mechanical and biological factors (Karsdal et al., 2014). Although a large number of clinical and animal studies have been conducted, the pathogenesis and progression of OA are not yet well understood. So, the basic purpose of OA treatment is to relieve symptoms, improve functions, and delay the process (McAlindon and Bannuru, 2018). Drug delivery in OA is a clinical challenge because of the specific avascular, dense, and occlusive tissue structure (Bijlsma et al., 2011). Based on the continuous development of conventional drugs (NSAIDS, glucosamine), the use of nanoparticles to deliver drugs for targeted therapy has contributed to a qualitative leap in enhancing drug penetration and sustained release in OA (Brown et al., 2019).

Osteoarthritis is often localized in specific joints, thus intra-articular (IA) injection is a more effective way to obtain therapeutic doses with minimal systemic side effects than systemic administration. However, the drug may be removed rapidly once it enters the joint, so a targeted delivery strategy can be more effective. Zheng’s team applied mangostemonin (FMN) as a therapeutic agent, a drug with extremely poor water solubility and low bioavailability, and prepared cartilage-targeting nanomicrospheres (PCFMN) by PEGylation of FMN followed by coupling with cartilage-targeting peptide (CollBP). Compared with FMN, PEGylation of FMN had higher drug solubility and CollBP increased drug accumulation in the joint site. A variety of inflammation-related factors decreased significantly after treatment, which also ameliorated ACLT-induced cartilage destruction, and ultimately achieved an effective OA retarding effect (Xiong et al., 2021). Xue et al. affixed a type II collagen-targeting peptide to a mesoporous polydopamine (MPDA) dual drug delivery system (RB@MPMW) modified with a metal organic backbone (MOF). After nanoparticle stimulation with a near-infrared (NIR) laser, bilirubin (Br) was released for rapid ROS scavenging, and rapamycin’s (Rap) release further boosted autophagy activation and chondrocyte protection (Figure 4C). The targeted release of both drugs at cartilage sites effectively delayed cartilage degeneration in the ACLT rat model (Xue S. et al., 2021).

### 3.3 Osteosarcoma

Osteosarcoma (OS) is a malignant bone tumor that is the most prevalent as primary sarcoma in kids and adolescents. The characteristics of aggressiveness, malignancy, and poor prognosis make it a serious threat to human health (Kansara et al., 2014). Early treatment of OS is based on amputation, which causes physical and psychological damage to patients; with the progress of medicine, the current treatment paradigm of OS is preoperative neoadjuvant chemotherapy, surgical resection, and postoperative adjuvant chemotherapy, and the 5 year survival rate of the disease has risen from 20% to about 60% (Isakoff et al., 2015; Gill and Gorlick, 2021). Whereas, the state of the clinical application suggests that the problems of tumor resistance, non-targeted drug delivery, and the high cost and side effects of chemotherapy have not improved the efficacy in essence, especially for patients with metastasis or recurrence (González-Fernández et al., 2017). With the era of precision medicine, the development of targeted drug delivery systems may be an effective means to raise survival rates (Jurek et al., 2017).

Chen’s team has developed two nanoplatforms for the targeted treatment of osteosarcoma. One is cisplatin (CDDP)-crosslinked hyaluronic acid nanogels loaded with DOX (CDDPHANG/DOX). CDDP not only acts as an anti-cancer drug but also serves as a crosslinking agent, which prevents premature drug release and more accumulation in the tumor. The second is calcium carbonate (CaCO_3_) crosslinked hyaluronic acid nanoparticles to deliver DOX, and calcium crosslinking also ensures the stability of the nanoparticles (Figure 4D). Both nanosystems exhibit sensitivity to the acidic tumor microenvironment, prolonged blood circulation time, and good biocompatibility (Zhang et al., 2018a; Zhang et al., 2018b).

In another study in which bisphosphonates were used as targeting ligands, the coupling of alendronate (ALN) with CD44’s ligand hyaluronic acid (HA) was affixed with DSPE PEG2K-COOH via a bioreduced disulfide bond (-SS-) to give the functionalized lipid ALN-HA-SS-L, which was linked to liposomes loaded with the anticancer drug DOX. *In vitro* experiments verified that the responsive liposomes released the drug after disassembled in glutathione-rich cancer cells, showing high cytotoxicity and a rapid cellular uptake rate against human OS MG-63 cells, and a significant growth inhibitory effect was observed in the *in situ* OS mouse model with an improved survival rate in mice (Feng et al., 2019). Overall, the assembled dual-targeting redox-sensitive liposomes for bone and CD44 showed promising results for OS.

## 4 Conclusion and Outlook

The increasing prevalence of bone diseases has received a great deal of attention. While conventional drug therapies can provide some relief, a series of limitations in drug delivery and adverse effects have also kept the research of bone diseases at bay. Finding low-toxicity, stable, and osteotropic compounds or carriers for targeted drug delivery is the key to the study of bone disease treatment. In recent years, many scholars have used targeted molecules to directly affix drugs to form pre-drugs that can specifically direct therapeutic drugs to the bone, but because of the inconsistent stability of the covalent bonding of pre-drugs, releasing drugs at the appropriate time remains a problem. In the last few decades, nanomedicine has been introduced which seems to solve this problem, mainly depending on the multifunctionality of nanomaterials, as nanoparticles can be loaded with drugs for bone-targeted therapeutics through functionalized modification of bone-targeting moieties or stimulatory response functional groups. Bone targeted delivery enables drugs to accumulate specifically in the diseased skeleton and target cells, improving the pharmacokinetics of the drug and enhancing therapeutic efficacy.

The current review summarizes systemic/local bone-targeting approaches and their application in bone metabolic diseases. Although these strategies have shown promising outcomes *in vivo* in orthopedic disease studies, it is rare that drug delivery nanosystems modified with bone-targeting moieties have been successfully translated into clinical applications. This is limited by the drug loading rate of the nanosystem, storage stability, ability to dissociate and release the drug, blood circulation, and *in vivo* metabolism; all of these issues still await further study. Even research has shown that most nanomaterials have minimal toxicity, and only a very small number of nanomedicines have been approved for marketing by the FDA, which suggests that we still need to further investigate their safety in order to make them safe for long-term use and achieve maximum clinical efficacy. Therefore, in future research, the effects of nanoparticles on cells need to be explored more comprehensively and systematically to improve drug loading and release, and bone-targeting mechanisms should also be explored further to find more specific targeting ligands. In addition, communication and cooperation between orthopedic surgeons and researchers should be strengthened to design nanosystems oriented to clinical problems.

Despite achieving clinical translation of bone-targeted therapies is still a long way off, there is no doubt that the development of bone-targeted drug delivery NPs is a highly promising research and these ongoing studies will offer a basis for further improvement of the properties and selectivity of these systems. We look forward to more ideal targeting ligands and carriers to be developed in the bone-targeting research efforts to make clinical applications of bone-targeting possible.
